# Use of Sentinel Surveillance Platforms for Monitoring SARS-CoV-2 Activity: Evidence From Analysis of Kenya Influenza Sentinel Surveillance Data

**DOI:** 10.2196/50799

**Published:** 2024-03-25

**Authors:** Daniel Owusu, Linus K Ndegwa, Jorim Ayugi, Peter Kinuthia, Rosalia Kalani, Mary Okeyo, Nancy A Otieno, Gilbert Kikwai, Bonventure Juma, Peninah Munyua, Francis Kuria, Emmanuel Okunga, Ann C Moen, Gideon O Emukule

**Affiliations:** 1 Influenza Division US Centers for Disease Control and Prevention Atlanta, GA United States; 2 Global Influenza Branch Influenza Division US Centers for Disease Control and Prevention Nairobi Kenya; 3 Centre for Global Health Research Kenya Medical Research Institute Kisumu Kenya; 4 Henry Jackson Foundation Nairobi Kenya; 5 Disease Surveillance and Response Unit Ministry of Health Nairobi Kenya; 6 National Influenza Centre Laboratory National Public Health Laboratories Ministry of Health Nairobi Kenya; 7 Directorate of Public Health Ministry of Health Nairobi Kenya

**Keywords:** SARS-CoV-2, COVID-19, influenza, sentinel surveillance, Kenya, epidemic, local outbreak, respiratory infection, surveillance, cocirculation, monitoring, respiratory pathogen

## Abstract

**Background:**

Little is known about the cocirculation of influenza and SARS-CoV-2 viruses during the COVID-19 pandemic and the use of respiratory disease sentinel surveillance platforms for monitoring SARS-CoV-2 activity in sub-Saharan Africa.

**Objective:**

We aimed to describe influenza and SARS-CoV-2 cocirculation in Kenya and how the SARS-CoV-2 data from influenza sentinel surveillance correlated with that of universal national surveillance.

**Methods:**

From April 2020 to March 2022, we enrolled 7349 patients with severe acute respiratory illness or influenza-like illness at 8 sentinel influenza surveillance sites in Kenya and collected demographic, clinical, underlying medical condition, vaccination, and exposure information, as well as respiratory specimens, from them. Respiratory specimens were tested for influenza and SARS-CoV-2 by real-time reverse transcription polymerase chain reaction. The universal national-level SARS-CoV-2 data were also obtained from the Kenya Ministry of Health. The universal national-level SARS-CoV-2 data were collected from all health facilities nationally, border entry points, and contact tracing in Kenya. Epidemic curves and Pearson *r* were used to describe the correlation between SARS-CoV-2 positivity in data from the 8 influenza sentinel sites in Kenya and that of the universal national SARS-CoV-2 surveillance data. A logistic regression model was used to assess the association between influenza and SARS-CoV-2 coinfection with severe clinical illness. We defined severe clinical illness as any of oxygen saturation <90%, in-hospital death, admission to intensive care unit or high dependence unit, mechanical ventilation, or a report of any danger sign (ie, inability to drink or eat, severe vomiting, grunting, stridor, or unconsciousness in children younger than 5 years) among patients with severe acute respiratory illness.

**Results:**

Of the 7349 patients from the influenza sentinel surveillance sites, 76.3% (n=5606) were younger than 5 years. We detected any influenza (A or B) in 8.7% (629/7224), SARS-CoV-2 in 10.7% (768/7199), and coinfection in 0.9% (63/7165) of samples tested. Although the number of samples tested for SARS-CoV-2 from the sentinel surveillance was only 0.2% (60 per week vs 36,000 per week) of the number tested in the universal national surveillance, SARS-CoV-2 positivity in the sentinel surveillance data significantly correlated with that of the universal national surveillance (Pearson *r*=0.58; *P*<.001). The adjusted odds ratios (aOR) of clinical severe illness among participants with coinfection were similar to those of patients with influenza only (aOR 0.91, 95% CI 0.47-1.79) and SARS-CoV-2 only (aOR 0.92, 95% CI 0.47-1.82).

**Conclusions:**

Influenza substantially cocirculated with SARS-CoV-2 in Kenya. We found a significant correlation of SARS-CoV-2 positivity in the data from 8 influenza sentinel surveillance sites with that of the universal national SARS-CoV-2 surveillance data. Our findings indicate that the influenza sentinel surveillance system can be used as a sustainable platform for monitoring respiratory pathogens of pandemic potential or public health importance.

## Introduction

Influenza can cause local outbreaks and seasonal epidemics and has pandemic potential, which makes it an important respiratory infection to monitor for prompt public health response [1]. Influenza occurs in multiple epidemics all year round in the tropics and mostly in winter or cold seasons in temperate regions [1,2]. Although influenza virus infection can be asymptomatic in about 30%-50% of infections or cause mild illness [3-5], in some people, influenza virus infection can cause severe illness leading to hospitalization, intensive care admission, mechanical ventilation, or death [1,6]. It is estimated that influenza is associated with 290,000 to 650,000 annual deaths globally [7,8]. Sub-Saharan Africa is one of the highest-burden regions [7,8], but influenza remained poorly monitored until recently when countries began building influenza surveillance systems to monitor circulating strains [2] and contribute to global data to inform strain selections for annual influenza vaccines [9,10].

In December 2019, an outbreak of severe respiratory illness caused by a novel coronavirus (SARS-CoV-2) was reported in Wuhan, China [11]. The World Health Organization declared SARS-CoV-2 infection that causes COVID-19 as a pandemic on March 11, 2020. Given that this outbreak occurred during the Northern Hemisphere influenza season, the public health community was concerned about the potential cocirculation of influenza and SARS-CoV-2 that could overburden health care systems. During the influenza months in winter, surges in medical visits and increase in health care use have been well documented [12]. Data from the Northern Hemisphere or countries with strong public health surveillance systems showed that influenza activity unexpectedly remained minimal during the COVID-19 pandemic [13-19]. Similarly, countries in the sub-Saharan African region, which has some of the highest burden of influenza, reported very minimal influenza activity [20]. However, given that most of these countries are still developing their influenza surveillance systems, it is unclear whether the minimal activity reported was due to a break in surveillance because of the response measures to the pandemic. In addition, little is known about how data from sentinel influenza surveillance platforms could be used to gauge SARS-CoV-2 circulation in sub-Saharan Africa.

Kenya, an equatorial country in sub-Saharan Africa, has implemented sentinel influenza surveillance since 2007 and developed a strong platform that continued to function during the COVID-19 pandemic. This provides an opportunity to understand influenza circulation in a tropical setting during the COVID-19 pandemic. Here, we describe influenza circulation and coinfection with SARS-CoV-2 in Kenya during the COVID-19 pandemic and compare SARS-CoV-2 virus detection from the influenza sentinel surveillance system with that of the universal national SARS-CoV-2 surveillance that included data from all facilities and border points in Kenya. The findings of this evaluation could inform the implementation or strengthening of national sentinel influenza systems and leveraging these platforms to monitor respiratory pathogens of pandemic potential.

## Methods

### Study Sites

**Figure 1:**
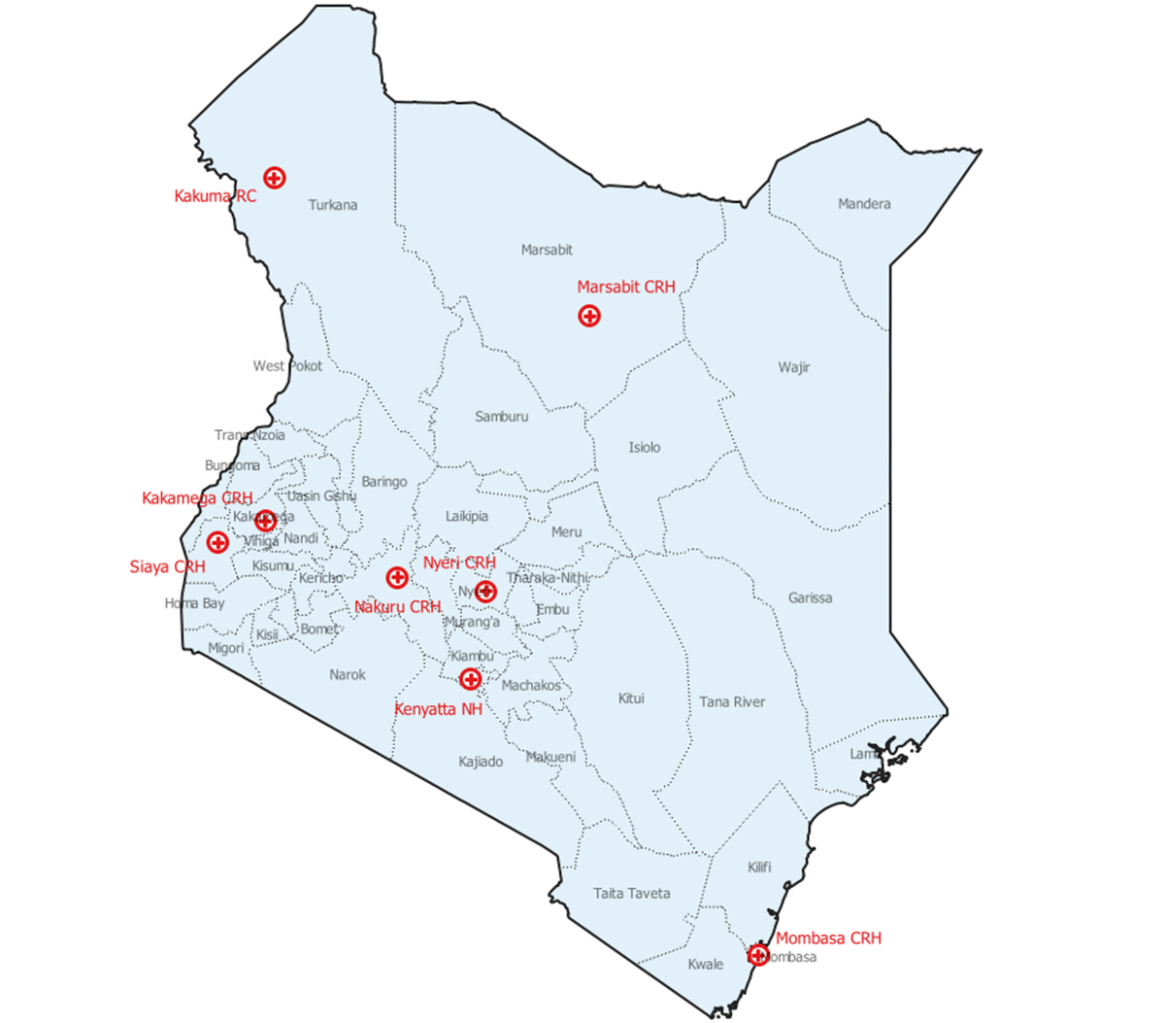
Sentinel surveillance sites for severe acute respiratory illness and influenza-like illness in Kenya, April 2020 to March 2022. CRH: County Referral Hospital; NH: National Hospital; RC: Refugee Camp.

The Kenya Ministry of Health (MoH), in collaboration with the US Centers for Disease Control and Prevention (CDC), established sentinel surveillance for influenza in selected health care facilities in 2007 [21]. Currently, there are 8 sentinel surveillance sites (Figure 1) located within 6 county-level referral hospitals (Coast General Teaching and Referral Hospital, Kakamega, Nakuru, Nyeri, Marsabit, and Siaya [22]), 1 tertiary referral hospital (Kenyatta National Hospital [23]), and the International Rescue Committee hospital in the Kakuma Refugee Camp. All sites enroll patients of all ages except the site in the Kenyatta National Hospital, which enrolls only pediatric cases.

### Surveillance Procedures

Trained surveillance officers (either a nurse or a clinical officer) identified and enrolled patients who met the severe acute respiratory illness (SARI) or influenza-like illness (ILI) case definitions [24]. We defined a SARI case as an acute onset of respiratory illness (within the last 10 days) with fever (reported or measured fever of ≥38 °C) and cough, requiring hospitalization. Eligible patients with SARI were identified and enrolled 5 days a week (Monday to Friday) within 48 hours of hospital admission for all sites except for Kakuma Refugee Camp, where enrollment was done Monday to Saturday. In addition, surveillance staff reviewed admission logbooks every Monday to identify and enroll any eligible patients with SARI admitted during the weekends. We defined ILI as an acute onset of respiratory illness (within the last 10 days) with measured fever of ≥38 °C and cough. The first 3 patients with ILI were enrolled per week at the outpatient clinics. Surveillance for SARS-CoV-2 was initiated and integrated into the routine surveillance for influenza among patients presenting with SARI or ILI (as defined earlier) in the sentinel sites in April 2020. This analysis used data collected from the sentinel sites (Figure 1) and the national SARS-CoV-2 surveillance data reported to the MoH from April 2020 through March 2022.

### Specimen Collection and Testing

Trained surveillance officers collected nasopharyngeal or nasal and oropharyngeal swabs from the enrolled patients. Nasopharyngeal or nasal and oropharyngeal swabs were placed in a single cryovial containing 3-mL sterile viral transport media and labeled with the patient’s unique identifier, a barcode number, and the date of specimen collection. Samples were stored at 2-8 °C at the sentinel surveillance sites for up to 36 hours and then shipped to the National Influenza Center laboratory in Nairobi, where each specimen was made into 3 aliquots and tested.

We tested an aliquot of each specimen for SARS-CoV-2 and influenza-viral ribonucleic acid at the CDC-supported Kenya Medical Research Institute laboratory and National Influenza Center laboratory, respectively, by real-time reverse transcription polymerase chain reaction (rtRT-PCR). Each assay had a target for *Homo sapiens* (human) RNase P gene for monitoring specimen quality. Influenza testing was done as previously described [21,22]. The primer and probe sequences for the influenza A, influenza B, and ribonuclease P gene targets were as per the approved CDC’s Human Influenza Virus Real-Time RT-PCR Detection and Characterization Panel [21,22]. All specimens positive for influenza type A were subtyped for H3, H5, and A(H1N1) pdm09 using rtRT-PCR. The SARS-CoV-2 laboratory testing protocol has been described elsewhere [25]. A cycle threshold (Ct) of <40.0 was considered positive and ≥40.0 or no amplification was considered negative.

### Data Collection and Variable Definitions

The surveillance staff enrolled patients using an electronic structured questionnaire with questions on demographics, clinical presentation, underlying medical conditions, medical history, vaccination history, and exposures. The questionnaire was administered to participants or their parents or legal guardians using a password-protected netbook. In addition, surveillance staff conducted a medical chart review to complete a follow-up exit questionnaire for patients with SARI. This review was done to abstract data on clinical outcomes of hospitalization. Laboratory data were recorded into Freezerworks software (Dataworks Development, Inc) and merged with epidemiological information. All data were stored in password-protected Microsoft Access or SQL databases on a central server at the Kenya Medical Research Institute, Center for Global Health Research campus in Nairobi.

We defined influenza virus or SARS-CoV-2 detection as a positive rtRT-PCR test of influenza (A or B) or SARS-CoV-2, respectively. A participant was assumed to have been coinfected if the respiratory sample was rtRT-PCR positive for both influenza (A or B) and SARS-CoV-2. Severe clinical illness was defined as any of oxygen saturation <90%, in-hospital death, admission to intensive care unit, mechanical ventilation, or a report of any danger sign (ie, inability to drink or eat, severe vomiting, grunting, stridor, or unconsciousness among children younger than 5 years).

### Kenya National SARS-CoV-2 Surveillance

In Kenya, the universal national SARS-CoV-2 surveillance was led by MoH focusing initially on points of entry and contact of people exposed to individuals who tested positive for SARS-CoV-2. Once there was evidence of community spread of SARS-CoV-2 infections in Kenya, all persons suspected to have COVID-19 or presenting with ILI in health facilities were tested for COVID-19 [26]. In addition, cargo vessel crew at all entry points were screened for SARS-CoV-2. Testing was initially limited to a few laboratories but later expanded to 96 public and private laboratories conducting rtRT-PCR tests and 472 conducting rapid diagnostic tests. All 8 influenza sentinel sites contributed data to the universal national SARS-CoV-2 surveillance throughout the study period.

### Statistical Analysis

Frequencies and percentages were calculated to describe the characteristics of participants overall and by patient type (in- or outpatients). Microsoft Excel (Microsoft Corp) charts were used to describe influenza or SARS-CoV-2 trends based on a calculated monthly percentage of influenza and SARS-CoV-2 positivity. Pearson correlation coefficient (*r*) was used to compare trends in the 3-week moving average of SARS-CoV-2 positivity from the influenza sentinel surveillance data with that of the data from the universal national SARS-CoV-2 surveillance.

Logistic regression was conducted to assess the association between coinfection and severe clinical illness among SARI cases. Univariate and multivariable logistic regression analyses were also conducted to examine factors associated with the detection of influenza (A or B) only, SARS-CoV-2 only, and influenza and SARS-CoV-2 coinfection among enrolled patients, stratified by ILI versus SARI cases. In all multivariable models, we included age, surveillance site, and in-patient or outpatient status a priori and any other variable of interest (underlying conditions, sex, smoker in household, hospitalization in past year, symptoms) that was significant at *P*<.05 in the univariate analysis. All analyses were performed using Stata Statistical Software (version 13.0; StataCorp).

### Ethical Considerations

The Kenya MoH determined the sentinel influenza surveillance to be a routine public health activity, and a nonresearch project determination was also received from CDC (project 0900f3eb81e74404). Thus, ethical review was not required to enroll and collect data from patients at the sentinel sites. We obtained informed verbal consent from participants or parents or legal guardians of children before the administration of questionnaires and collection of specimens. In addition to verbal consent of parents or guardians, children aged 7 years and older provided assent before participation.

## Results

### Demographic and Clinical Characteristics

Patient characteristics are summarized in Table 1. From April 2020 through March 2022, we enrolled 7349 patients from the sentinel influenza surveillance sites in Kenya. Most (n=5606, 76.3%) of the patients were children aged younger than 5 years. The median age of all the enrolled patients was 1.5 (IQR 0.7-4.6) years. Of all the enrolled patients, 3331 (45.3%) were female participants, and 899 (12.2%) had at least one underlying medical condition (eg, HIV positive: n=171, 2.3%; heart disease: n=281, 3.8%; chronic neurological or neuromuscular disease: n=185, 2.5%; asthma: n=223, 3%; diabetes: n=81, 1.1%; and other: n=93, 1.3%). Overall, 5932 (80.7%) were patients with SARI, while the rest were patients with ILI who were seen as outpatients. The Kakuma site provided the highest percentage of enrolled patients (n=2316, 31.5%), followed by Siaya County Referral Hospital with 18.1% (n=1332). Kakamega County Referral Hospital had the least number of enrolled patients (n=174, 2.4%). Overall, 46% (n=2727) of inpatients (SARI cases) presented with severe clinical illness. Among patients presenting with severe clinical illness, 20.9% (n=1238) were admitted to the intensive care unit, 8.9% (n=527) had oxygen saturation <90%, 2.3% (n=134) were put on mechanical ventilation during admission, 41% (2084/5080) were children younger than 5 years who had at least one danger sign reported, and 5.4% (n=319) died during admission (Table 1).

**Table 1:** Characteristics of participants at enrollment, among those seen as outpatients or inpatients, in the 8 influenza sentinel surveillance sites in Kenya, April 2020 to March 2022.

Characteristics			Outpatients (n=1417)		Inpatients (n=5932)		All (N=7349)
Age (years), median (IQR)			14.0 (2.4-32.9)		1.2 (0.6-2.7)		1.5 (0.7-4.6)
**Age groups, n (%)**							
	0-11 months	159 (11.2)		2627 (44.3)		2786 (37.9)	
	12-23 months	135 (9.5)		1312 (22.1)		1447 (19.7)	
	2-4 years	232 (16.4)		1141 (19.2)		1373 (18.7)	
	5-14 years	198 (14)		348 (5.9)		546 (7.4)	
	15-64 years	658 (46.4)		365 (6.2)		1023 (13.9)	
	≥65 years	35 (2.5)		139 (2.3)		174 (2.4)	
	<5 years	526 (37.1)		5080 (85.6)		5606 (76.3)	
	≥5 years	891 (62.9)		852 (14.4)		1743 (23.7)	
	<13 years	677 (47.8)		5418 (91.3)		6095 (82.9)	
	≥13 years	740 (52.2)		514 (8.7)		1254 (17.1)	
Female, n (%)			711 (50.2)		2620 (44.2)		3331 (45.3)
**Site name, n (%)**							
	Kenyatta National Hospital	5 (0.4)		1053 (31.1)		1058 (14.4)	
	Coast General Teaching and Referral Hospital	11 (0.8)		446 (7.5)		457 (6.2)	
	Nyeri County Referral Hospital	291 (20.5)		506 (8.5)		797 (10.9)	
	Nakuru County Referral Hospital	26 (1.8)		916 (15.4)		942 (12.8)	
	Kakamega County Referral Hospital	14 (1)		160 (2.7)		174 (2.4)	
	Siaya County Referral Hospital	512 (36.1)		820 (13.8)		1332 (18.1)	
	Marsabit Community Referral Hospital	85 (6)		188 (3.2)		273 (3.7)	
	Kakuma Refugee Camp	473 (33.4)		1843 (31.1)		2316 (31.5)	
**Underlying medical condition, n (%)**			189 (13.3)		710 (12)		899 (12.2)
	HIV infection	47 (3.3)		124 (2.1)		171 (2.3)	
	HIV unknown	467 (33)		837 (14.1)		1304 (17.7)	
	Heart disease	61 (4.3)		220 (3.7)		281 (3.8)	
	Chronic neurological or neuromuscular disease	14 (1)		171 (2.9)		185 (2.5)	
	Asthma	64 (4.5)		159 (2.7)		223 (3)	
	Diabetes	18 (1.3)		63 (1.1)		81 (1.1)	
	Other^a^	18 (1.3)		75 (1.3)		93 (1.3)	
Hospitalized in the past 12 months, n (%)			73 (5.2)		810 (13.7)		883 (12)
Current smoker in the household, n (%)			39 (2.8)		258 (4.4)		297 (4)
**SARS-CoV-2, n (%)**							
	Tested	1400 (98.8)		5799 (97.8)		7199 (98)	
	Positive	235 (16.8)		533 (9.2)		768 (10.7)	
**Influenza, n (%)**							
	Tested	1402 (98.9)		5822 (98.2)		7224 (98.3)	
	Influenza positive	103 (7.4)		526 (9)		629 (8.7)	
	Influenza A positive	47 (45.6)		259 (49.2)		306 (48.7)	
	Influenza A (H3N2) positive	39 (83)		238 (92)		277 (90.5)	
	Influenza A (H1N1) pdm09 positive	0 (0)		3 (1.2)		3 (1)	
	Not subtyped or could not be subtyped	8 (17)		18 (7)		26 (8.5)	
	Influenza B positive	59 (57.3)		278 (52.9)		337 (53.6)	
**SARS-CoV-2 and influenza, n (%)**							
	Tested for both	1390 (98.1)		5775 (97.4)		7165 (97.5)	
	SARS-CoV-2 and influenza positive	9 (0.7)		54 (0.9)		63 (0.9)	
	SARS-CoV-2 and influenza A (H3N2) positive	3 (33.3)		27 (50)		30 (47.6)	
	SARS-CoV-2 and influenza A or not subtyped or could not be subtyped	0 (0)		1 (1.9)		1 (1.6)	
	influenza B positive	6 (66.7)		23 (42.6)		29 (46)	
	SARS-CoV-2 and A (H3N2) and influenza B positive	0 (0)		3 (5.6)		3 (4.8)	
**Severe clinical illness, n (%)^b^**			198 (14)		2727 (46)		2925 (39.8)
	Oxygen saturation <90%	28 (2)		527 (8.9)		555 (7.6)	
	In-hospital death	N/A^c^		319 (5.4)		319 (4.3)	
	Admission to ICU^d^ or HDU^e^	N/A		1238 (20.9)		1238 (16.9)	
	Mechanical ventilation during admission	N/A		134 (2.3)		134 (1.8)	
	Presence of a danger sign^f^	181^g^ (34.4)		2084^h^ (41)		2265^i^ (40.4)	

^a^Any of liver disease, renal disease, and cancer.

^b^Severe clinical illness was defined as any of oxygen saturation <90%, in-hospital death, admission to intensive care unit or high dependence unit, mechanical ventilation, or a report of any danger sign among children younger than 5 years (inability to drink or eat, severe vomiting, grunting, stridor, or unconsciousness).

^c^N/A: not applicable.

^d^ICU: intensive care unit.

^e^HDU: high dependence unit.

^f^Report of any of unable to drink or breastfeed at all, vomits everything, grunting, stridor, and unconscious (comatose and not awake) among children younger than 5 years.

^g^n=526.

^h^n=5080.

^i^n=5606.

### Influenza and SARS-CoV-2 Circulation

We tested 7199 (98%) specimens for SARS-CoV-2, 7224 (98.3%) specimens for influenza, and 7165 (97.5%) of the specimens for both influenza and SARS-CoV-2. We detected influenza (A or B) in 8.7% (n=629); 7.4% (n=103) tested positive among outpatients and 9% (n=526) among inpatients. Of the patients with influenza, 48.7% (n=306) had influenza A, while 53.6% (n=337) had influenza B. Among the influenza A cases, 90.5% (n=277) were A (H3N2), 1% (n=3) A (H1N1) pdm09, and 8.5% (n=26) not subtyped. Among patients who were tested for SARS-CoV-2, 10.7% (n=768) tested positive; 16.8% (235/1400) tested positive among outpatients and 9.2% (533/5799) among inpatients (Table 1).

**Figure 2:**
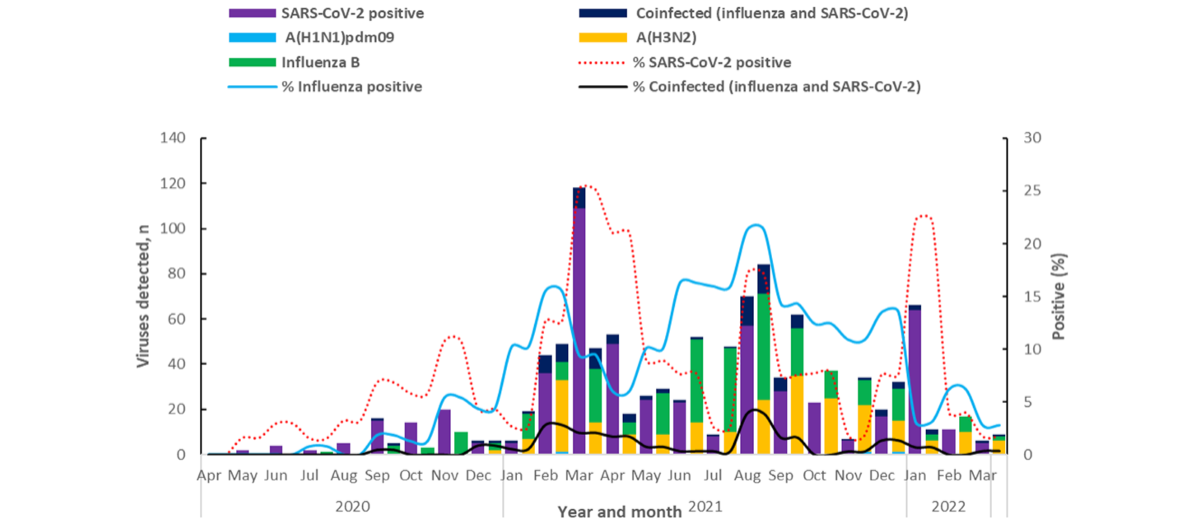
Monthly trends of influenza activity (including circulating subtypes), SARS-CoV-2 activity, and coinfections of influenza and SARS-CoV-2 using data from the 8 influenza sentinel surveillance sites in Kenya, April 2020 to March 2022. From April 2020 through March 2022, influenza cocirculated with SARS-CoV-2, with influenza positivity rates sometimes surpassing that of SARS-CoV-2. Data source: Kenya influenza sentinel surveillance system.

Detection of both viruses continued throughout the evaluation period, with influenza positivity remaining higher than SARS-CoV-2 positivity from mid-May 2021 through late December 2021. The highest influenza positivity was 21.4% (71/333) in August 2021 when SARS-CoV-2 positivity was 17.2% (57/333). The highest peak of SARS-CoV-2 positivity was in March 2021 at 25.1% (109/434) when influenza positivity was 9.4% (41/434; Figure 2).

### SARS-CoV-2 Positivity in the Influenza Sentinel Surveillance Versus the Universal National SARS-CoV-2 Surveillance Data

**Figure 3:**
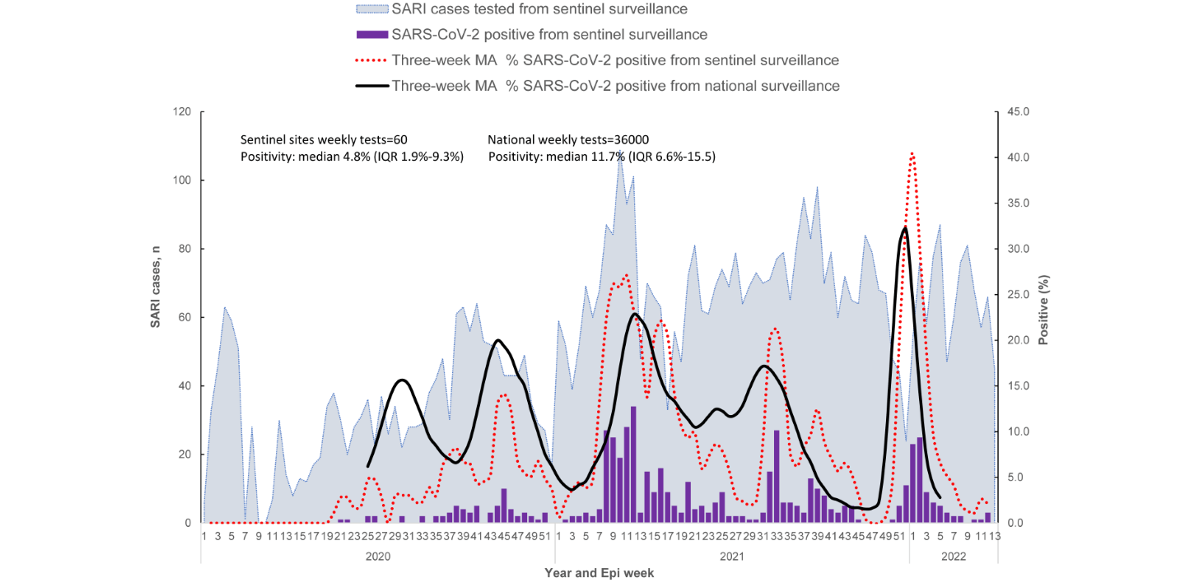
Three-week MA of SARS-CoV-2 positivity using data from the 8 influenza sentinel surveillance sites in Kenya versus universal national SARS-CoV-2 surveillance data from all tests conducted in Kenya, April 2020 to March 2022. This figure shows number of specimens tested from the sentinel sites and 3-week MA of SARS-CoV-3 positivity from the sentinel sites and the Kenya national SARS-CoV-2 surveillance data. SARS-CoV-2 detection and peak from the sentinel surveillance, with a median of 60 (IQR 38-71) weekly tests, significantly correlated (Pearson r=0.58; *P*<.001) with the detection and peak activity from the Kenya universal national SARS-CoV-2 surveillance data, which had a median of 36,000 (IQR 28,000-43,000) weekly tests. Epi: epidemiology; MA: moving average; SARI: severe acute respiratory infection.

SARS-CoV-2 positivity in the data collected from the sentinel sites, with a median of 60 (IQR 38-71) weekly tests during the study period, significantly correlated (Pearson *r*=0.58; *P*<.001) with SARS-CoV-2 positivity in the Kenya universal national SARS-CoV-2 surveillance data, which had a median of 36,000 (IQR 28,000-43,000) weekly tests (Figure 3).

### Coinfection and Severe Clinical Illness

We detected influenza and SARS-CoV-2 coinfection in 0.9% (63/7165) of those who were tested for both infections. Thus, 8.2% (63/765) of those who tested positive for SARS-CoV-2 also tested positive for influenza. The odds ratios of clinical severe illness among participants with coinfection were similar to those of patients with influenza only (adjusted odds ratio [aOR] 1.06, 95% CI 0.72-1.39) and SARS-CoV-2 only (aOR 1.00, 95% CI 0.54-2.10) infection (Table 2).

**Table 2:** Association between influenza and SARS-CoV-2 coinfection and severe clinical illness among patients hospitalized with severe acute respiratory illness (SARI) from 8 influenza sentinel surveillance sites in Kenya (n=1004), April 2020 to March 2022.^a^

Variable			Had severe illness,^b^ n (%)		uOR^c^ (95% CI)		aOR^d^ (95% CI)
**Detection**							
	SARS-CoV-2 and influenza A or B coinfection	20 (4.9)		Reference		Reference	
	Influenza A or B	190 (46.2)		1.23 (0.69-2.21)		0.91 (0.47-1.79)	
	SARS-CoV-2	201 (48.9)		1.15 (0.64-2.05)		0.92 (0.47-1.82)	
**Age group**							
	0-11 months	188 (45.7)		1.35 (0.91-1.98)		1.07 (0.61-1.85)	
	12-23 months	84 (20.4)		0.82 (0.53-1.25)		0.67 (0.37-1.20)	
	2-4 years	66 (16.1)		0.71 (0.46-1.11)		0.57 (0.31-1.04)	
	5-12 years	13 (3.2)		0.56 (0.27-1.15)		0.25 (0.10-0.62)	
	≥13 years	60 (14.6)		Reference		Reference	
**Any underlying medical condition**							
	No	332 (80.8)		Reference		Reference	
	Yes	79 (19.2)		1.75 (1.23-2.48)		N/A^e^	
**Heart disease**							
	No	42 (10.2)		Reference		Reference	
	Yes	369 (89.8)		2.48 (1.50-4.12)		1.44 (0.76-2.73)	
**Diabetes**							
	No	9 (2.2)		Reference		Reference	
	Yes	402 (97.8)		1.08 (0.45-2.60)		N/A	
**Gastroenteritis or diarrhea**							
	No	112 (27.3)		Reference		Reference	
	Yes	299 (72.8)		1.74 (1.29-2.36)		1.22 (0.84-1.77)	
**Duration of SARI symptoms at presentation**							
	1-3 days	379 (92.2)		Reference		Reference	
	≥4 days	32 (7.8)		0.92 (0.58-1.46)		N/A	

^a^Odds ratios were adjusted for age, site of data collection, and any variable that was significant at *P*<.05 in the univariate analysis.

^b^Severe clinical illness was defined as any of oxygen saturation <90%, in-hospital death, admission to intensive care unit or high dependence unit, mechanical ventilation, or a report of any danger sign among children younger than 5 years (inability to drink or eat, severe vomiting, grunting, stridor, or unconsciousness).

^c^uOR: unadjusted odds ratio.

^d^aOR: adjusted odds ratio.

^e^N/A: Not applicable.

### Factors Associated With Influenza and SARS-CoV-2 Infection Detection Among SARI and ILI Cases

None of the variables included in the multivariable analysis were associated with influenza-only detection or influenza and SARS-CoV-2 coinfection among SARI cases. However, detection of SARS-CoV-2 only was significantly higher in patients aged 13 years and older compared to those aged 0-11 months (aOR 3.85, 95% CI 1.86-8.02; Multimedia Appendix 1). Adjusting for other covariates, influenza-only detection was less common in patients with ILI diagnosed with malaria (aOR 0.09, 95% CI 0.03-0.27). Among ILI cases, SARS-CoV-2–only detection was associated with age group, malaria diagnosis, and presenting with chills (Multimedia Appendix 2).

## Discussion

### Summary of Main Findings

In this study, we found that influenza continued to circulate in Kenya throughout the COVID-19 pandemic. Although the number of weekly tests in the influenza surveillance system was only 0.2% (60 per week vs 36,000 per week) of the number of tests conducted in the universal national surveillance for SARS-CoV-2, we found that peaks in SARS-CoV-2 positivity from our sentinel surveillance sites coincided with peaks in SARS-CoV-2 positivity from the universal national SARS-CoV-2 surveillance data. Our analysis further revealed that about 1 in 12 patients who tested positive for SARS-CoV-2 also tested positive for influenza; however, we did not find any significant evidence of increased severe clinical illness among these patients compared to those who tested positive for influenza only or SARS-CoV-2 only.

### Influenza and SARS-CoV-2 Cocirculation

Multiple reports from elsewhere [13,14,17,19,27] indicated that influenza activity was minimal and far below expectation at the height of the SARS-CoV-2 pandemic from 2020 through 2021. Our results suggest that unlike these countries, Kenya continued to record substantially high levels of influenza activity during the pandemic. Some experts have attributed the minimal influenza activity observed in many countries during the SARS-CoV-2 pandemic to the impacts of widespread mitigation measures such as social distancing, facial covering, and stay-at-home measures [15,16,18,28]. However, Kenya implemented mitigation measures with similar stringency to those of countries that recorded minimal influenza activity such as South Africa and the United States (Multimedia Appendix 3) [29]. Although our data are limited to explain why Kenya experienced high influenza activity despite implementing some of the stringent mitigation measures, our results suggest the importance of local surveillance of influenza and other pathogens of public health importance and pandemic potential. Because of geographic and sociocultural differences, data from one country may not be applicable to another country.

### Influenza Sentinel Surveillance System Versus Universal National SARS-CoV-2 Surveillance

We observed that the detection rates and peak activity of SARS-CoV-2 from the sentinel surveillance were well aligned with that of the universal national surveillance, suggesting that the data from the sentinel system was able to detect increased SARS-CoV-2 activity during the study period. This is consistent with findings reported from Indonesia, in which SARS-CoV-2 trends in the influenza surveillance data aligned with COVID-19 national surveillance data [30]. When the pandemic started, the Kenyan government, like many other countries, implemented several ad hoc systems to respond to the pandemic, including opening border surveillance units and national influenza surveillance coordination centers and increasing laboratory capacity to collect SARS-CoV-2 data and implement interventions to control the pandemic. Although it might be good to identify all cases to respond appropriately, a universal national surveillance system is labor- and resource-intensive and not sustainable in the long term in Kenya and other settings. Therefore, there is a need for a sustainable approach for ongoing monitoring of SARS-CoV-2 and other pathogens of pandemic potential in general to detect early signals and implement effective interventions. Our results support the World Health Organization’s [31] recommendation for integrated surveillance of SARS-CoV-2 and influenza and suggest that influenza sentinel surveillance systems can serve this purpose for routine monitoring and provide signals of increased activity of SARS-CoV-2 to aid the development of appropriate public health interventions and decisions. These findings provide further evidence about the need to implement and sustain a strong influenza surveillance system in all countries, especially, those in sub-Saharan Africa.

### Influenza and SARS-CoV-2 Coinfection and Severe Clinical Illness

Besides concerns about health systems being overwhelmed by influenza and SARS-CoV-2 cocirculation, one major concern about this cocirculation was the potential for coinfection with both viruses and consequent severe outcomes in patients. Our understanding of the extent and potential impact of influenza and SARS-CoV-2 coinfection on clinical illness is still evolving. A few studies have reported varying results regarding the prevalence and severity of coinfection [32-36]. Prevalence of influenza coinfection in people with SARS-CoV-2 as low as 0.6% has been reported in the United States [35] and as high as 12.6% has been reported in Guangzhou, China [33]. In this study, we found 8.2% (63/768) influenza coinfection in participants who tested positive for SARS-CoV-2. Unlike previous findings from animal models [32] and UK adults [36], but consistent with findings from India [34], we did not find a significant increase in the frequency of severe clinical presentation among these individuals compared to those infected with only influenza or only SARS-CoV-2. Our results may have been impacted by the age composition of the study sample. The majority were children, who are already at increased risk of severe influenza outcomes [37] and often have less severe COVID-19 [38]. Our findings together with the existing literature on influenza and SARS-CoV-2 coinfection suggest a need to continue to monitor cases of coinfection and their clinical presentations and outcomes to inform preventive measures and clinical management.

### Limitations

Although we used data from a good influenza sentinel surveillance system that collects data from all ages and our analysis controlled for multiple potential confounders, there are some limitations of the study. The criteria used to define severe clinical illness were in-hospital presentations and outcomes and did not include outcomes outside the sentinel site hospitals such as death prior to admission or after discharge. Therefore, more studies are needed to examine disease severity among people with influenza and SARS-CoV-2 coinfection. The age of 65 years and older is a risk factor for severe outcomes of SARS-CoV-2 infection; however, this population is underrepresented in this analysis (139/5932, 2.3%), so we cannot draw inferences on coinfections for this population. Most of the participants were children younger than 13 years of age; therefore, we could not assess differences among older patients. Data used in this study may not be representative of the Kenyan population because the sites were within only selected counties that do not necessarily represent the rest of the country.

### Conclusions

Unlike many other countries, influenza significantly cocirculated with SARS-CoV-2 during the early weeks of 2020 through the first quarter of 2022 in Kenya, and SARS-CoV-2 positivity data from the influenza sentinel surveillance sites were comparable to that of the national surveillance system. Our findings reinforce the need to implement or strengthen national influenza sentinel surveillance systems to monitor respiratory diseases of public health importance or those with pandemic potential. Influenza or respiratory disease sentinel surveillance sites could be used as a sustainable platform for national monitoring of SARS-CoV-2 activity.

## Appendix

Multimedia Appendix 1 Factors associated with influenza and SARS-CoV-2 infection detection among patients hospitalized with severe acute respiratory illness from 8 influenza sentinel surveillance sites in Kenya (n=1004), April 2020 to March 2022.

Multimedia Appendix 2 Factors associated with influenza and SARS-CoV-2 infection detection among outpatients with influenza-like illness from 8 influenza sentinel surveillance sites in Kenya (n=1004), April 2020 to March 2022.

Multimedia Appendix 3 Kenya implemented SARS-CoV-2 mitigation measures with similar stringency to those of countries that recorded minimal influenza activity such as South Africa and the United States.

## Abbreviations

aOR: adjusted odds ratio
CDC: Centers for Disease Control and Prevention
ILI: influenza-like illness
MoH: Ministry of Health
rtRT-PCR: real-time reverse transcription polymerase chain reaction
SARI: severe acute respiratory illness

## Data Availability

The data sets generated and analyzed during this study are not publicly available because they were collected for public health surveillance by the Kenya Ministry of Health but are available from RK (focal person for influenza surveillance, division of disease surveillance and response, Kenya Ministry of Health) on reasonable request.
