# Supplementary material for: Use of Sentinel Surveillance Platforms for Monitoring SARS-CoV-2 Activity: Evidence From Analysis of Kenya Influenza Sentinel Surveillance Data
Source: JMIR Public Health Surveill. 2024 Mar 25;10:e50799. doi: 10.2196/50799 (PMC11002741; doi:10.2196/50799)
Supplement: Multimedia Appendix 1 [file publichealth_v10i1e50799_app1.docx]

**Multimedia Appendix 1. Factors associated with influenza and SARS-CoV-2 infection detection among patients hospitalized with severe acute respiratory illness from 8 influenza sentinel surveillance sites in Kenya (n=1004), April 2020 to March 2022.**

|  | **Influenza (A or B) (n=5775)** | | **SARS-CoV-2 (n=5775)** | | **Influenza (A or B) and SARS-CoV-2 co-detection (n=5775)** | |
| --- | --- | --- | --- | --- | --- | --- |
|  | **uOR (95% CI)** | **aOR (95% CI)** | **uOR (95% CI)** | **aOR (95% CI)** | **uOR (95% CI)** | **aOR (95% CI)** |
| Age in years (n, %) | |  |  |  |  |  |
| 0-11 months | Ref | Ref | Ref | Ref | Ref | Ref |
| 12-23 months | **1.59 (1.26-2.00)** | 1.48 (0.79-2.77) | 0.96 (0.75-1.22) | 0.59 (0.23-1.51) | **2.15 (1.03-4.47)** | 1.45 (0.59-3.55) |
| 2-4 years | **1.52 (1.20-1.93)** | 1.44 (0.81-2.58) | 0.96 (0.74-1.24) | 0.96 (0.47-1.95) | 1.97 (0.91-4.27) | 1.36 (0.52-3.53) |
| 5-12 years | 1.16 (0.76-1.76) | 1.16 (0.58-2.34) | 0.72 (0.45-1.15) | 1.01 (0.45-2.25) | 0.56 (0.07-4.28) | 0.56 (0.07-4.72) |
| ≥13 years | **1.47 (1.06-2.03)** | 1.43 (0.72-2.87) | **2.88 (2.23-3.73)** | **3.85 (1.86-8.02)** | **4.44 (2.04-9.66)** | 2.42 (0.82-7.15) |
| Sex (n, %) | |  |  |  |  |  |
| Male | Ref | Ref | Ref | Ref | Ref | Ref |
| Female | 1.16 (0.97-1.39) | - | 1.07 (0.89-1.28) | - | 1.27 (0.74-2.17) | - |
| Current smoker in the household (n, %) | **1.49 (1.02-2.19)** | 1.12 (0.62-2.00) | 0.80 (0.50-1.29) | - | 2.28 (0.90-5.77) | - |
| Hospitalization in the last 12 months | 1.10 (0.85-1.42) | - | 1.25 (0.98-1.60) | - | 1.45 (0.73-2.89) | - |
| Had an underlying medical condition, n (%)** | 1.08 (0.82-1.41) | - | **1.64 (1.29-2.09)** | - | 1.68 (0.84-3.35) | - |
| HIV-infected vs HIV-uninfected  HIV-unknown vs HIV-Uninfected | 0.50 (0.22-1.15)  0.91 (0.70-1.18) | -  - | 0.82 (0.43-1.59)    0.59 (0.44-0.81) | -  - | 1.57 (0.38-6.50)    **0.17 (0.02 - 0.84)** | 2.18 (0.45-10.53)  0.71 (0.09-5.48) |
| Heart disease (yes vs no) | **1.72 (1.16-2.54)** | 1.80 (0.96-3.36) | **2.42 (1.70-3.44)** | 1.36 (0.77-2.40) | 2.07 (0.74-5.79) | - |
| Chronic neurological or neuromuscular disease | 0.84 (0.47-1.49) | - | **1.60 (1.02-2.50)** | 1.94 (0.83-4.51) | 1.29 (0.31-5.34) | - |
| Asthma | 1.16 (0.69-1.97) | - | 0.91 (0.51-1.61) | - | NE | - |
| Diabetes | 1.52 (0.72-3.21) | - | **4.24 (2.43-7.40)** | 1.23 (0.60-2.52) | **10.3 (3.96-26.83)** | 2.83 (0.79-10.07) |
| Other^c^ | 1.85 (0.97-3.54) | - | 1.82 (0.95-3.49) | - | 1.52 (0.21-11.17) | - |
| Clinical diagnosis (n, %) | | | |  |  |  |
| Pneumonia | 1.00 (0.80-1.24) | - | **1.39 (1.09-1.76)** | 1.35 (0.89-2.07) | 1.32 (0.64-2.71) | - |
| Acute respiratory distress syndrome | **0.19 (0.07-0.50)** | 7.19 (0.94-55.3) | 1.08 (0.68-1.71) | - | NE | - |
| Bronchitis | 0.84 (0.51-1.40) | - | 1.36 (0.89-2.07) | - | NE | - |
| Malaria | 0.94 (0.70-1.28) | - | **0.63 (0.44-0.89)** | 0.51 (0.23-1.13) | 0.69 (0.25-1.93) | - |
| Sepsis | **0.66 (0.44-0.98)** | 1.43 (0.67-3.08) | 0.89 (0.62-1.27) | - | 1.13 (0.45-2.85) | - |
| Malnutrition | 0.96 (0.78-1.18) | - | **0.75 (0.59-0.93)** | 0.48 (0.23-1.03) | 0.71 (0.36-1.42) | - |
| Gastroenteritis/diarrhea | 0.83 (0.67-1.04) | - | 0.95 (0.77-1.18) | - | 0.84 (0.43-1.63) | - |
| Anemia | **0.65 (0.48-0.88)** | 0.44 (0.20-0.98) | 0.63 (0.46-0.86) | - | 0.25 (0.06-1.03) | - |
| Clinical presentation (n, %) | | |  |  |  |  |
| Difficulty in breathing | 0.94 (0.77-1.16) | - | 1.09 (0.88-1.35) | - | **0.56 (0.32-0.98** | 0.55 (0.27-1.08) |
| Chest pain | 1.17 (0.87-1.55) | - | **1.50 (1.12-2.01)** | 0.71 (0.45-1.11) | 0.73 (0.32-1.66) | - |
| Chills | 0.91 (0.72-1.15) | - | 0.96 (0.76-1.22) | - | **0.35 (0.12-0.98)** | 0.59 (0.19-1.85) |
| Diarrhea | 0.82 (0.62-1.06) | - | 0.97 (0.76-1.24) | - | 0.76 (0.35-1.65) | - |
| Rhinorrhea | **1.28 (1.07-1.55)** | 1.10 (0.72-1.69) | 0.85 (0.71-1.01) | - | 0.90 (0.53-1.55) | - |
| Sore muscles | 1.01 (0.72-1.41) | - | 1.29 (0.93-1.80) | - | 1.02 (0.45-2.28) | - |
| Sore throat | **1.46 (1.06-2.02)** | 1.06 (0.69-1.66) | **1.70 (1.24-2.34)** | 0.78 (0.51-1.20) | 1.65 (0.69-3.91) | - |
| Vomiting | 1.01 (0.84-1.21) | - | 0.83 (0.69-1.00) | **-** | 0.92 (0.53-1.60) | - |
| Wheezing | 1.08 (0.83-1.40) | - | 0.99 (0.76-1.29) | - | 0.83 (0.35-1.94) | - |

uOR, unadjusted odds ratio; aOR, adjusted odds ratio; CI, confidence interval. Odds ratios were adjusted for age, site of data collection, patient type, and any variable that was significant at p<0.05 in the univariate analysis; NE, not estimated for lack of outcome data

** Not included in the adjusted model to avoid collinearity:
