# Supplementary material for: Use of Sentinel Surveillance Platforms for Monitoring SARS-CoV-2 Activity: Evidence From Analysis of Kenya Influenza Sentinel Surveillance Data
Source: JMIR Public Health Surveill. 2024 Mar 25;10:e50799. doi: 10.2196/50799 (PMC11002741; doi:10.2196/50799)
Supplement: Multimedia Appendix 2 [file publichealth_v10i1e50799_app2.docx]

**Multimedia Appendix 2. Factors associated with influenza and SARS-CoV-2 infection detection among outpatients with influenza-like illness from 8 influenza sentinel surveillance sites in Kenya (n=1004), April 2020 to March 2022.**

|  | **Influenza (A or B) (n=1390)** | | **SARS-CoV-2 (n=1390)** | | **Influenza (A or B) and SARS-CoV-2 co-detection (n=1390)** | |
| --- | --- | --- | --- | --- | --- | --- |
|  | **uOR (95% CI)** | **aOR (95% CI)** | **uOR (95% CI)** | **aOR (95% CI)** | **uOR (95% CI)** | **aOR (95% CI)** |
| Age in years (n, %) | |  |  |  |  |  |
| 0-11 months | Ref | Ref | Ref | Ref | Ref | Ref |
| 12-23 months | 1.18 (0.54-2.58) | 0.95 (0.31-2.95) | 0.70 (0.28-1.74) | NE | 1.16 (0.07-18.79) | 1.08 (0.07-17.92) |
| 2-4 years | 1.33 (0.67-2.64) | 0.93 (0.33-2.60) | 0.73 (0.34-1.61) | **0.18 (0.07-0.47)** | 1.40 (0.13-15.56) | 1.27 (0.11-14.56) |
| 5-12 years | 0.97 (0.44-2.14) | 2.09 (0.72-6.08) | 1.14 (0.52-2.51) | **0.39 (0.20-0.76)** | 1.05 (0.06-16.89) | 1.22 (0.07-20.71) |
| ≥13 years | 0.54 (0.28-1.02) | 1.14 (0.41-3.20) | **3.78 (2.09-6.83)** | NE | 0.87 (0.10-7.82) | 0.90 (0.09-9.30) |
| Sex (n, %) | |  |  |  |  |  |
| Male | Ref | Ref | Ref | Ref | Ref | Ref |
| Female | 0.86 (0.57-1.29) | - | 1.06 (0.80-1.41) | - | 0.49 (0.12-1.98) | - |
| Current smoker in the household (n, %) | 1.04 (0.32-3.44) | - | 0.72 (0.28-1.87) | - | NE | - |
| Hospitalization in the last 12 months | 1.63 (0.76-3.51) | - | 0.71 (0.35-1.45) | - | NE | - |
| Had an underlying medical condition, n (%)** | **0.39 (0.17-0.89)** | NE | **1.51 (1.03-2.21)** | - | NE | - |
| HIV-infected vs HIV-uninfected  HIV-unknown vs HIV-Uninfected | 0.50 (0.20-2.11)  0.91 (0.70-1.18) | -  - | **2.12 (1.07-4.20)**  **1.57 (1.17-2.11)** | 0.79 (0.30-1.21)  0.80 (0.31-2.04) | NE  0.56 (0.12-2.70) | - |
| Heart disease (yes vs no) | 0.42 (0.10-1.76) | - | **1.54 (0.83-2.85)** | 0.61 (0.30-1.21) | NE | - |
| Chronic neurological or neuromuscular disease | NE | - | **2.79 (0.93-8.42)** | 1.46 (0.35-6.12) | NE | - |
| Asthma | 0.64 (0.20-2.07) | - | 0.63 (0.28-1.41) | - | NE | - |
| Diabetes | 0.73 (0.10-5.55) | - | **4.07 (1.59-10.42)** | 2.02 (0.69-5.94) | NE | **-** |
| Other^c^ | NE | - | 1.15 (0.32-4.06) | - | NE | - |
| Clinical diagnosis (n, %) | | | |  |  |  |
| Pneumonia | 0.61 (0.24-1.54) | - | **2.52 (1.63-3.92)** | **1.97 (1.15-3.36)** | 1.55 (0.19-12.52) | - |
| Malaria | **0.24 (0.11-0.52)** | **0.09 (0.03-0.27)** | 0.69 (0.43-1.08) | 0.85 (0.49-1.48) | 1.30 (0.18-9.29) | - |
| Malnutrition | 1.19 (0.46-3.04) | - | 0.46 (0.18-1.16) | 0.53 (0.23-1.22) | NE | - |
| Gastroenteritis/diarrhea | 1.13 (0.53-2.41) | - | 1.06 (0.62-1.82) | - | NE | - |
| Clinical presentation (n, %) | | |  |  |  |  |
| Difficulty in breathing | 1.17 (0.76-1.80) | - | 1.27 (0.94-1.72) | - | 3.10 (0.83-11.59) | - |
| Chest pain | 1.21 (0.74-2.00) | - | **1.94 (1.42-2.64)** | 1.23 (0.87-1.76) | **9.81 (1.18-81.77)** | 1.53 (0.33-7.21) |
| Chills | 0.71 (0.47-1.07) | - | **1.52 (1.14-2.03)** | **1.57 (1.06-2.32)** | 1.41 (0.38-5.29) | - |
| Diarrhea | 1.19 (0.55-2.58) | - | 0.79 (0.45-1.37) | - | NE | - |
| Rhinorrhea | **1.84 (1.19-2.86)** | 1.31 (0.64-2.68) | 0.98 (0.74-1.30) | - | 1.45 (0.36-5.84) | - |
| Sore muscles | 0.32 (0.10-1.09) | - | 1.30 (0.74-2.28) | - | 1.63 (0.29-9.04) | - |
| Sore throat | 1.39 (0.83-2.31) | - | **1.93 (1.41-2.64)** | 1.15 (0.81-1.63) | 2.13 (0.39-11.64) | - |
| Vomiting | **1.56 (1.02-2.39)** | 0.94 (0.47-1.89) | **0.36 (0.24-0.55)** | **0.39 (0.22-0.69)** | NE | - |
| Wheezing | 0.93 (0.36-2.35) | - | 1.09 (0.59-2.03) | - | NE | - |

uOR, unadjusted odds ratio; aOR, adjusted odds ratio; CI, confidence interval. Odds ratios were adjusted for age, site of data collection, patient type, and any variable that was significant at p<0.05 in the univariate analysis; NE, not estimated for lack of outcome data

** Not included in the adjusted model to avoid collinearity
