# Supplementary figures and images for: Use of Sentinel Surveillance Platforms for Monitoring SARS-CoV-2 Activity: Evidence From Analysis of Kenya Influenza Sentinel Surveillance Data
Source: JMIR Public Health Surveill. 2024 Mar 25;10:e50799. doi: 10.2196/50799 (PMC11002741; doi:10.2196/50799)

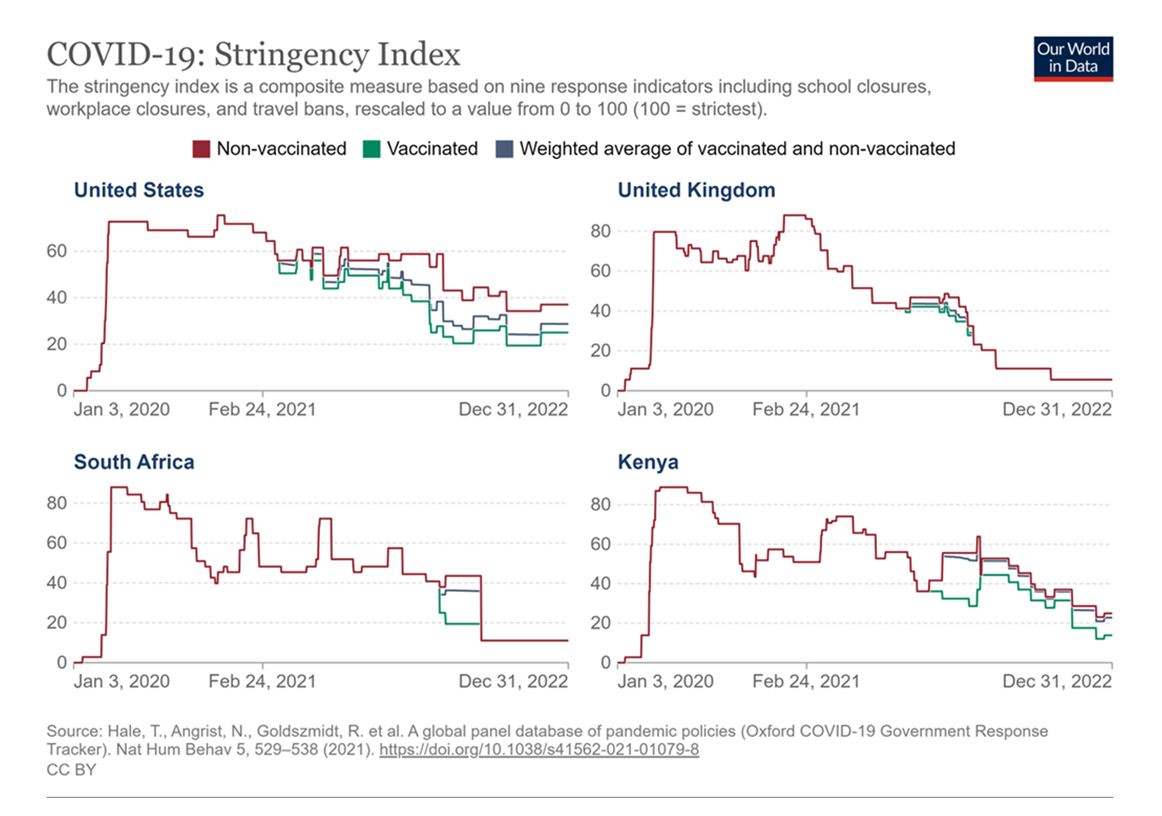

Supplement: Multimedia Appendix 3 [file publichealth_v10i1e50799_app3.png]
